# Hydatid liver cyst causing portal vein thrombosis and cavernous transformation: a case report and literature review 

**Published:** 2016

**Authors:** Serdar Kirmizi, Cuneyt Kayaalp, Sezai Yilmaz

**Affiliations:** *Inonu University, Liver Transplantation Institute, Malatya, Turkey *

**Keywords:** Echinococcus granulosus, portal hypertension, portal shunt, liver surgery, percutaneous drainage, biliary fistula

## Abstract

A 33-year-old male with abdominal distention after meals was admitted to the hospital. He had a history of surgery for hydatid liver cyst. The cyst was located at the liver hilum and there were portal venous thrombosis and cavernous transformation. It had been treated with partial cystectomy, omentoplasty and albendazole. Two years later at the admission to our center, his laboratory tests were in normal ranges. Abdominal imaging methods revealed splenomegaly, portal vein thrombosis, cavernous transformation and the previously operated hydatid liver cyst. Upper gastrointestinal endoscopy demonstrated esophageal and gastric fundal varices. Due to his young age and low risk for surgery, the patient was planned for surgical treatment of both pathologies at the same time. At laparotomy, hydatid liver cyst was obliterated with omentum and there was no sign of active viable hydatid disease. A meso-caval shunt with an 8 mm in-diameter graft was created. In the postoperative period, his symptoms and endoscopic varices were regressed. There were four similar cases reported in the literature. This one was the youngest and the only one treated by a surgical shunt. Hydatid liver cysts that located around the hilum can lead to portal vein thrombosis and cavernous thrombosis. Treatment should consist of both hydatid liver cyst and portal hypertension. To the best of our knowledge, this was the first case of surgically treated portal vein thrombosis that was originated from a hydatid liver cyst.

## Introduction

 Hydatid disease, caused by Echinococcus granulosus, still continues to be a health problem in a significant part of the world. It is frequently located (60-70%) in the liver and if not treated properly, it may cause complications. The most frequent complication is cysto-biliary communication that can lead to hydatid jaundice or biliary fistula (1). Another fearsome complication is free abdominal perforation that may show a large scale of symptoms from a light abdominal pain (2) to anaphylaxis or shock (3). Here, we aimed to present a very rare complication of a hydatid liver cyst. 

## Case report

**Figure 1:**
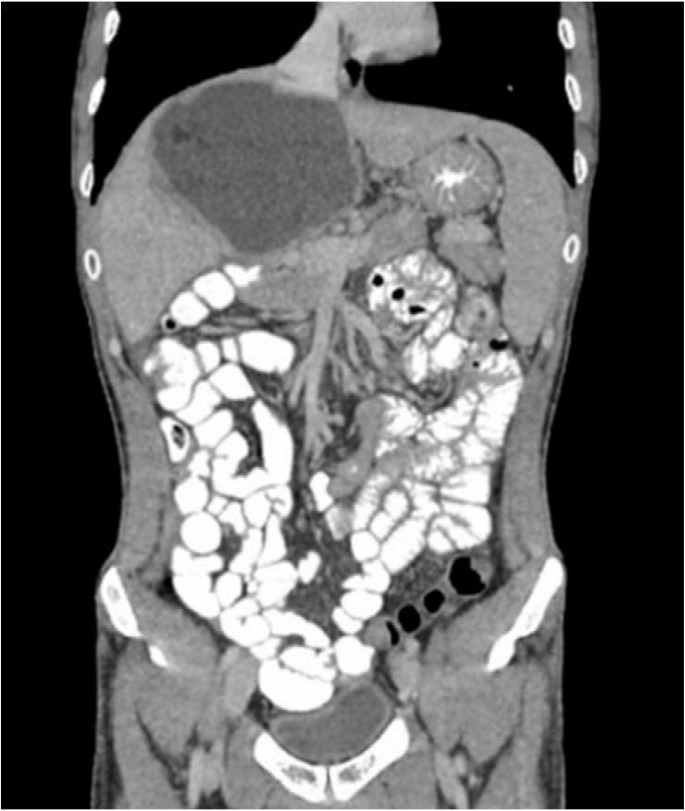
Hydatid cyst at the hepatic hilum, the cavernous transformation of the portal vein and patent superior mesenteric vein

A 33-year-old male was admitted for abdominal distention after meals. He had a history of surgery for hydatid liver cyst two years ago at another center. The 14x10 cm in size cyst was located at the hilum of the liver and there was portal venous thrombosis and cavernous transformation at hepatic hilum. The cyst had been treated with partial cystectomy, omentoplasty, and postoperative adjuvant albendazole (800mg per day). Two weeks after the surgery, a percutaneous drainage had been performed due to ongoing postoperative abdominal pain. After percutaneous drainage, a biliary fistula appeared and was treated with endoscopic retrograde cholangiography. At the admission to our center, his laboratory tests were in normal ranges. Abdominal imaging methods (ultrasound, computed tomography and magnetic resonance imaging) revealed splenomegaly, portal vein thrombosis, and cavernous transformation at hepatic hilum and the previously treated hydatid cyst (Figure 1).

**Figure 2:**
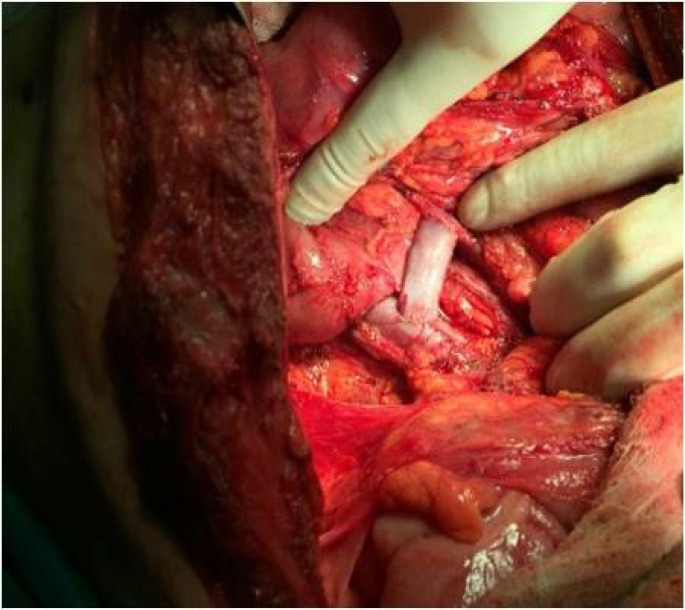
H-type meso-caval shunt between the superior mesenteric vein and vena cava

Upper gastrointestinal endoscopy demonstrated the esophageal and gastric fundal varices. Due to his young age and low risk for surgery, the patient was planned for a meso-caval shunt and hydatid liver cyst surgery. At laparotomy, hydatid liver cyst was obliterated with omentum. There was no sign of active viable hydatid disease. An 8 mm cryopreserved iliac vein graft was placed between the vena cava and the superior mesenteric vein (Figure 2). 

**Figure 3:**
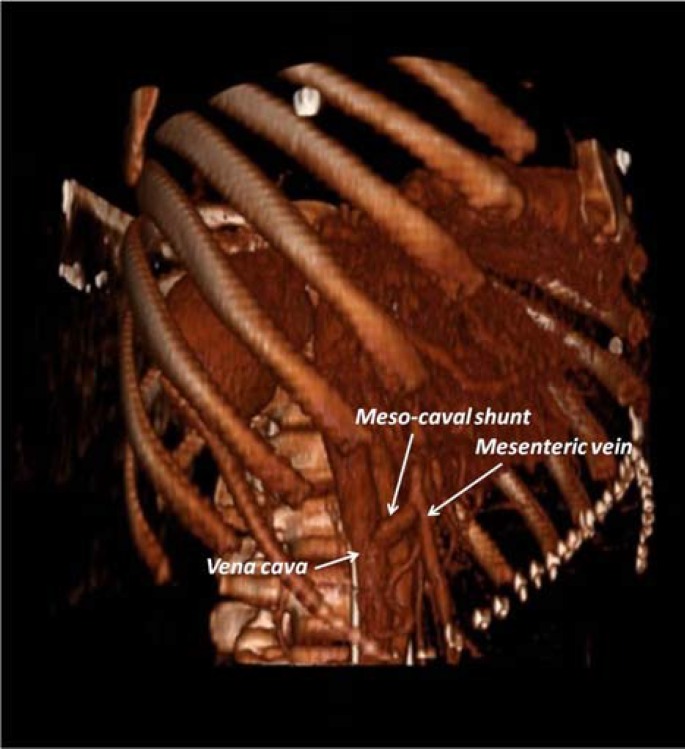
Postoperative radiological view of shunt

Cholecystectomy was also added for the prevention of the potential future surgeries at the hepatic hilum. Postoperative period was smooth except an intraabdominal oozing due to anticoagulant therapy. It was conservatively treated and the patient was discharged on day 10. The postoperative imaging evaluation showed the patent meso-caval shunt (Figure 3). In the postoperative period, his symptoms and endoscopic varices were regressed. 

## Discussion

Extra hepatic portal hypertension with the portal vein thrombosis is usually accompanied with portal cavernous transformation, which can be described as the neo-formation of venous collaterals around the occluded portal vein. Cavernous transformation occurs in the process of time as a secondary portal venous formation to convey the mesenteric blood to the liver. 

These compensatory venous collaterals usually cannot completely resolve the portal hypertension symptoms and its complications. Additionally, these veins ourselves can cause local compression on the extra hepatic biliary tract resulting to extra hepatic colestasis (4). Cavernous transformation is formed within 3 months after the initial pathology affecting the hepatic hilum (4). If the primary pathology persists, compensatory neovascularization and fibrotic reactions creates some irreversible anatomic and physiological changing around the hilum of the liver. The reason of portal vein thrombosis and cavernous transformation is various and the most frequent reasons in adults are cirrhosis and diseases leading to thrombosis (infection or hypercoagulation) (5-8).

Portal vein thrombosis and the cavernous transformation is a rare complication of hydatid liver cysts. There have been total four cases reported yet and all these hydatid cysts were located close to the hilum of the liver (Table 1) (5-8). Previously reported cases were all from endemic areas of echinococcosis, such as Spain (5), Turkey (6), Greece (7) and Chile (8). Three of all the cases were males (60%) and three of the cases (60%) had concomitant cysto-biliary communications (one occult and two evident cysto-biliary communications as hydatid jaundice). It is known that hiler location is an independent risk factor for cysto-biliary communication for hydatid liver cysts (9). Patients with hydatid liver cysts and portal cavernous transformation require treatments for both hydatid cysts and portal hypertension. In the previously reported four cases, treatment of hydatid cysts was done with surgery only in one case. Another two patients were treated with albendazole, and the last case was treated with endoscopic retrograde cholangiography. No treatment for portal hypertension was done in three cases and only one case was treated with propranolol. The mean ages of those four cases were 71 (ranged 62-84) and old age with co-morbidities could be the reason of conservative treatment approach in these cases. Our case was the youngest patient (33 year-old) in the literature and had a long life expectancy. We preferred a more active treatment modality for the prevention of potential complications of portal hypertension, the risks of portal ductopathy of the cavernous transformation and the hydatid liver cyst (4). 

**Table 1:** Literature review of the cases with hydatid liver cysts causing portal vein thrombosis and cavernous transformation.

No	#1	#2	#3	#4	#5
Author	Gil Egea (5)	Kayacetin (6)	Spanou (7)	Moisan (8)	Kirmizi
Year	1998	2004	2006	2012	2015
Country	Spain	Turkey	Greece	Chile	Turkey
Age	84	63	74	62	33
Gender	Female	Male	Male	Female	Male
Location	Hepatic hilum	Hepatic hilum	Hepatic hilum	Hepatic hilum	Hepatic hilum
Treatment of hydatid cyst	Albendazole	Surgery	ERCP	Albendazole	Surgery
Tretament of portal hypertension	None	None	None	Propranolol	Surgery

Despite the normal liver functions, patients with extra hepatic portal hypertension still have the risk of variceal bleeding and the risks of hypersplenism. Drugs decreasing the portal venous pressure, endoscopic band ligation/sclerotherapy for varices or percutaneous transjugular intrahepatic portosystemic shunt (TIPS) procedures are efficient to treat the portal hypertension. However, surgical shunts are still one of the most effective ways to avoid the variceal bleeding (10). There are several types of surgical shunts to decompress the portal pressure. If there was a portal vein thrombosis and cavernous transformation it precludes a shunt between the portal vein and the other veins. Then the branches of the portal vein can be used to compose meso-caval or spleno-renal shunts. Here, we did not prefer a spleno-renal shunt due to the technical difficulties of extensive venous collaterals around the splenic vein. An H-type meso-caval shunt can significantly decrease the portal vein pressure. To construct the H-type meso-caval shunt, we used a cryopreserved vein graft in a diameter 8 mm to decrease the risk of postoperative hepatic encephalopathy. There was no hepatic encephalopathy in our patient in the follow-up. 

In conclusion, hydatid liver cysts located around the hilum can result to portal vein thrombosis and cavernous thrombosis. Treatment should consist of both hydatid liver cyst and portal hypertension.
